# Supplementary material for: A Split-Marker System for CRISPR-Cas9 Genome Editing in Methylotrophic Yeasts
Source: Int J Mol Sci. 2023 May 3;24(9):8173. doi: 10.3390/ijms24098173 (PMC10179152; doi:10.3390/ijms24098173)
Supplement: Supplementary file 1 [file ijms-24-08173-s001.zip › ijms-2364300-supplementary.pdf]

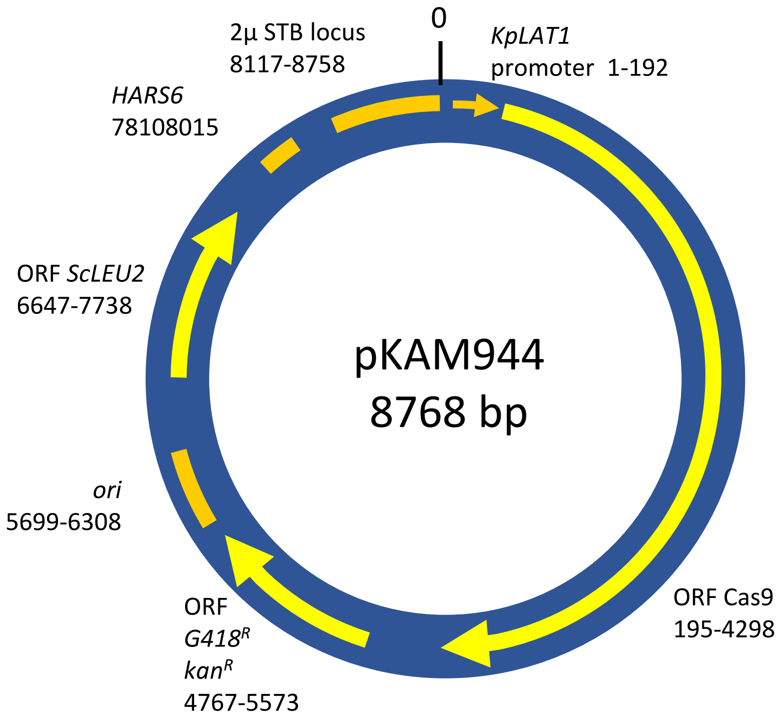


```
>pKAM944
ctagtttgagccgcttcaattgcggcaagtccccagttttgtttggtactcccctctcgcctcgcgtgtttttgttcctccgaattgtgcatcaccctgtcacatccgtacaccatcggaacactctgctcctcctcctttctcctcttaatt
gaacgtatatctaattaccctaataacaagtatcttcaaccatggacaagaagtactccattgggctcgatatcggcacaaacagcgtcggttgggccgtcattacggacgagtacaaggtgccgagcaaaaaattcaaagttctgggcaata
ccgatcgccacagcataaagaagaacctcattggcgccctcctgttcgactccggggagacggccgaagccacgcggctcaaaagaacagcacggcgcagatatacccgcagaaagaatcggatctgctacctgcaggagatctttagtaatg
agatggctaaggtggatgactctttcttccataggctggaggagtcctttttggtggaggaggataaaaagcacgagcgccacccaatctttggcaatatcgtggacgaggtggcgtaccatgaaaagtacccaaccatatatcatctgagga
agaagcttgtagacagtactgataaggctgacttgcggttgatctatctcgcgctggcgcatatgatcaaatttcggggacacttcctcatcgaggggggacctgaacccagacaacagcgatgtcgacaaactctttatccaactggttcaga
cttacaatcagcttttcgaagagaacccgatcaacgcatccggagttgacgccaaagcaatcctgagcgctaggctgtccaaatcccggcggctcgaaaacctcatcgcacagctccctggggagaagaagaacggcctgtttggtaatctta
tcgccctgtcactcgggctgacccccaactttaaatctaacttcgacctggccgaagatgccaagcttcaactgagcaaagacacctacgatgatgatctcgacaatctgctggcccagatcggcgaccagtacgcagaccttttttggcgg
caaagaacctgtcagacgccattctgctgagtgatattctgcgagtgaacacggagatcaccaaagctccgctgagcgctagtatgatcaagcgctatgatgagcaccaccaagacttgactttgctgaaggcccttgtcagacagcaactgc
ctgagaagtacaaggaaattttcttcgatcagtctaaaaatggctacgccggatacattgacggcggagcaagccaggaggaattttacaaatttattaagcccatcttggaaaaaatggacggcaccgaggagctgctggtaaagcttaaca
gagaagatctgttgcgcaaacagcgcactttcgacaatggaagcatcccccaccagattcacctgggcgaactgcacgctatcctcaggcggcaagaggatttctacccctttttgaaagataacagggaaaagattgagaaaatcctcacat
ttcggataccctactatgtaggcccccctcgcccggggaaattccagattcgcgtggatgactcgcaaatcagaagagactatcactccctggaacttcgaggaagtcgtggataagggggcctctgcccagtccttcatcgaaaggatgacta
actttgataaaaatctgcctaacgaaaaggtgcttcctaaacactctctgctgtacgagtacttcacagtttataacgagctcaccaaggtcaaatacgtcacagaagggatgagaaagccagcattcctgtctggagagcagaagaaagcta
tcgtggacctcctcttcaagacgaaccggaaagttaccgtgaaacagctcaaagaggactatttcaaaaagattgaatgtttcgactctgttgaaatcagcggagtggaggatcgcttcaacgcatccctgggaacgtatcacgatctcctga
aaatcattaaagacaaggacttcctggacaatgaggagaacgaggacattcttgaggacattgtcctcacccttacgttgtttgaagatagggagatgattgaagaacgcttgaaaacttacgctcatctcttcgacgacaaagtcatgaaac
agctcaagaggcgccgatatacaggatgggggcggctgtcaagaaaactgatcaatgggatccgagacaagcagagtggaaagacaatcctggattttcttaagtccgatggatttgccaaccggaacttcatgcagttgatccatgatgact
ctctcacctttaaggaggacatccagaaagcacaagtttctggccaggggggacagccttcacgagcacatcgctaatcttgcaggtagcccagctatcaaaaagggaatactgcagaccgttaaggtcgtggatgaactcgtcaaagtaatgg
gaaggcataagcccgagaatatcgttatcgagatggcccgagagaaccaaactacccagaagggacagaagaacagtagggaaaggatgaagaggattgaagagggtataaaagaactggggtcccaaatccttaaggaacacccagttgaaa
acacccagcttcagaatgagaagctctacctgtactacctgcagaacggcagggacatgtacgtggatcaggaactggacatcaatcggctctccgactacgacgtggatcatatcgtgccccagtcttttctcaaagatgattctattgata
ataaagtgttgacaagatccgataaaaatagagggaagagtgataacgtcccctcagaagaagttgtcaagaaaatgaaaaattattggcggcagctgctgaacgccaaactgatcacacaacggaagttcgataatctgactaaggctgaac
gaggtggcctgtctgagttggataaagccggcttcatcaaaaggcagcttgttgagacacgccagatcaccaagcacgtggcccaaattctcgattcacgcatgaacaccaagtacgatgaaaatgacaaactgattcgagaggtgaaagtta
ttactctgaagtctaagctcgtctcagatttcagaaaggactttcagttttataaggtgagagagatcaacaattaccaccatgcgcatgatgcctacctgaatgcagtggtaggcactgcacttatcaaaaaatatcccaagcttgaatctg
aatttgtttacggagactataaagtgtacgatgttaggaaaatgatcgcaaagtctgagcaggaaataggcaaggccaccgctaagtacttcttttacagcaatattatgaatttttcaagaccgagattacactggccaatggagagattc
ggaagcgaccacttatcgaaacaaacggagaaacaggagaaatcgtgtgggacaagggtagggatttcgcgacagtccggaaggtcctgtccatgccgcaggtgaacatcgttaaaaagaccgaagtacagaccggaggcttctccaaggaaa
gtatcctcccgaaaaggaacagcgacaagctgatcgcacgcaaaaaagattgggaccccaagaaatacggcggattcgattctcctacagtcgcttacagtgtactggttgtggccaaagtggagaaagggaagtctaaaaaactcaaaagcg
tcaaggaactgctgggcatcacaatcatggagcgatcaagcttcgaaaaaaaccccatcgactttctcgaggcgaaaggatataaagaggtcaaaaaagacctcatcattaagcttcccaagtactctctctttgagcttgaaaacggccgga
aacgaatgctcgctagtgcgggcgagctgcagaaaggtaacgagctggcactgccctctaaatacgttaatttcttgtatctggccagccactatgaaaagctcaaagggtcccccgaagataatgagcagaagcagctgttcgtggaacaac
acaaacactaccttgatgagatcatcgagcaaataagcgaattctccaaaagagtgatcctcgccgacgctaacctcgataaggtgctttctgcttacaataagcacagggataagcccatcagggagcaggcagaaaacattatccacttgt
ttactctgaccaacttgggcgcgcctgcagccttcaagtacttcgacaccaccatagacagaaagcggtacacctctacaaaggaggtcctggacgccacactgattcatcagtcaattacggggctctatgaaacaagaatcgacctctctc
agctcggtggagacagcagggctgaccccaagaagaagaggaaggtgtgagcttcgtccgacggcggcccgacgggtccgaggctgaagcttcgtacgctgcaggtcgacaacccttaatataacttcgtataatgtatgctatacgaagtta
ttaggtctctggcacgacaggtttcccgactggaaagcgggcagtgagcgcaacgcaattaatgtgagttagctcactcattaggcaccccaggctttacactttatgcttccggctcgtatgttgtgtggaattgtgagcggataacaattt
cacacaggaaacagcggcccggagtagtcatacgtgtaggtctttggcgtgatgaaaatcaggtggcgcacgacttttcgtaaagttcgggagggagtgctgcaaacggtatataaggaccagtttttctcgcacattatcaattgctcttta
gtacaaagataatatagggtgttatgagccatattcaacgggaaacgtcgaggccgcgattaaattccaacatggatgctgatttatatgggtataaatgggctcgcgataatgtcgggcaatcaggtgcgacaatctatcgcttgtatggga
agcccgatgcgccagagttgtttctgaaacatggcaaaggtagcgttgccaatgatgttacagatgagatggtcagactaaactggctgacggaatttatgcctcttccgaccatcaagcattttatccgtactcctgatgatgcatggttac
tcaccactgcgatccccggaaaaacagcattccaggtattagaagaatatcctgattcaggtgaaaatattgttgatgcgctggcagtgttcctgcgccggttgcattcgattcctgtttgtaattgtccttttaacagcgatcgcgtatttc
gtctcgctcaggcgcaatcacgaatgaataacggtttggttgatgcgagtgattttgatgacgagcgtaatggctggcctgttgaacaagtctggaaagaaatgcataaacttttgccattctcaccggattcagtcgtcactcatggtgatt
tctcacttgataaccttatttttgacgaggggaaattaataggttgtattgatgttggacgagtcggaatcgcagaccgataccaggatcttgccatcctatggaactgcctcggtgagttttctccttcattacagaaacggctttttcaaa
aatatggtattgataatcctgatatgaataaattgcagtttcatttgatgctcgatgagtttttctaatcagaattggttaattggttgtaacactggcagagcattacgctgacttgacgggacggcgcaagctcatgaccaaaatccctta
acgtgagttttcgttccactgagcgtcagaccccgtagaaaagatcaaaggatcttcttgagatcctttttttctgcgcgtaatctgctgcttgcaaacaaaaaaaccaccgctaccagcggtggtttgtttgccggatcaagagctaccaac
tctttttccgaaggtaactggcttcagcagagcgcagataccaaatactgttcttctagtgtagccgtagttaggccaccacttcaagaactctgtagcaccgcctacatacctcgctctgctaatcctgttaccagtggctgctgccagtgg
cgataagtcgtgtcttaccgggttggactcaagacgatagttaccggataaggcgcagcggtcgggctgaacggggggttcgtgcacacagcccagcttggagcgaacgacctacaccgaactgagatacctacagcgtgagctatgagaaag
cgccacgcttcccgaagggagaaaggcggacaggtatccggtaagcggcagggtcggaacaggagagcgcacgagggagcttccagggggaaacgcctggtatctttatagtcctgtcgggtttcgccacctctgacttgagcgtcgatttt
gtgatgctcgtcaggggggcggagcctatggaaaaacgccagcaacgcggcctttttacggttcctggccttttgctggccttttgctcacatgacattaacctataaaaataggcgtatcacgaggccctttcgtcttcaagaattaactgt
gggaatactcaggtatcgtaagatgcaagagttcgaatctcttagcaaccattatttttttcctcaacataacgagaacacacaggggcgctatcgcacagaatcaaattcgatgactggaaatttttttgttaatttcagaggtcgcctgacg
catataccttttcaactgattacatttcagcaatatatatatatatttcaaggatataccattctaatgtctgcccctaagaagatcgtcgttttgccaggtgaccacgttggtcaagaaatcacagccgaagccattaaggttcttaaagc
tatttctgatgttcgttccaatgtcaagttcgatttcgaaaatcatttaattggtggtgctgctatcgatgctacaggtgtcccacttccagatgaggcgctggaagcctccaagaaggttgatgccgttttgttaggtgctgtgggtggtcc
taaatggggtacaggtagtgttagacctgaacaaggtttactaaaaatccgtaaagaacttcaattgtacgccaacttaagaccatgtaactttgcatccgactctcttttagacttatctccaatcaagccacaatttgctaaaggtactga
cttcgttgttgtcagagaattagtgggaggtatttactttggtaagagaaaggaagacgatggtgatggtgtcgcttgggatagtgaacaatacaccgttccagaagtgcaaagaatcacaagaatggccgctttcatggccctacaacatga
gccaccattgcctatttggtccttggataaagctaatgttttggcctcttcaagattatggagaaaaactgtggaggaaaccatcaagaacgaatttcctacattgaaggttcaacatcaattgattgattctgccgccatgatcctagttaa
gaacccaacccacctaaatggtattatcatcaccagcaatatgtttggtgacatcatctccgatgaagcctccgttatcccaggttccttgggtttgttgccatctgcgtccttggcctctttgccagacaagaacaccgcatttggtttgta
cgaaccatgccacggttctgctccagatttgccaaagaataaggtcaaccctatcgccactatcttgtctgctgcaatgatgttgaaattgtcattgaacttgcctgaagaaggtaaggccattgaagatgcagttaaaaaggttttggatgc
aggtatcagaactggtgatttaggtggttccaacagtaccaccgaagtcggtgatgctgtcgccgaagaagttaagaaaatccttgcttaaaaagattctcttttttatgatatttgtaccgggccccccctcgaggtcgatcttaggtttt
gtagttaccggtcgctgtttgatggctatttttatttttgagtttttaataattgtggtaatttttttgaaggtcgcagcaaccatttctggctctgaaaaggctacaacatatgaccggggccatttagtaatcagcacaattcccttcta
taatatcaagactcaatgcaactgctagtattaccttcgaatagcaggaattttccgatatccatatggttctcagcctagcagaaatgtcaccaaataagacttcgtggccaagctggtttaaggcatgcgactgttaatcgcaagatcgac
ggtatcggtagtatatagagacaaaatagaagaaaccgttcataattttctgaccaatgaagaatcatcaacgctatcactttctgttcacaaagtatgcgcaatccacatcggtatagaatataatcggggatgcctttatcttgaaaaaat
gcacccgcagcttcgctagtaatcagtaaacgcgggaagtggagtcaggcttttttatggaagagaaaatagacaccaaagtagccttcttctaaccttaacggacctacagtgcaaaaagttatcaagagactgcattatagagcgcacaa
aggagaaaaaaagtaatctaagatgctttgttagaaaaatagcgctctcgggatgcatttttgtagaacaaaaaagaagtatagattctttgttggtaaaatagcgctctcgcgttgcatttctgttctgtaaaaatgcagctcagattcttt
gtttgaaaaattagcgctctcgcgttgcatttttgttttacaaaaatgaagcacagattcttcgttggtaaaatagcgctttcgcgttgcatttctgttctgtaaaaatgcagctcagattctttgtttgaaaaattagcgctctcgcgttgc
atttttgttctacaaaatgaagcacagatgcttcgttatcagatcca
```

**Figure S1**. Map and 5'-3' sequence of the pKAM944 plasmid

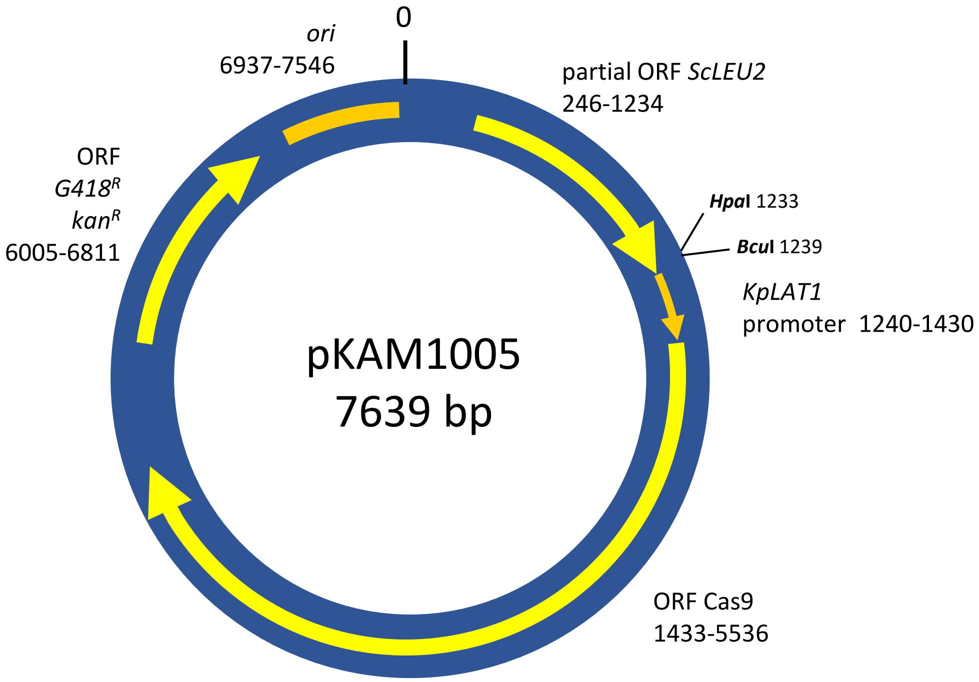


>pKAM1005
cctttcgtcttcaagaattaactgtgggaatactcaggtatcgtaagatgcaagagttcgaatctcttagcaaccattatttttttcctcaacataacgagaacacacaggggcgctatcgcacagaatcaaattcgatgactggaaatttt
ttgttaatttcagaggtcgcctgacgcatatacctttttcaactgattacatttcagcaatatatatatatatttcaaggatataccattctaatgtctgcccctaagaagatcgtcgttttgccaggtgaccacgttggtcaagaaatcac
agccgaagccattaaggttcttaaagctatttctgatgttcgttccaatgtcaagttcgatttcgaaaatcatttaattggtggtgctgctatcgatgctacaggtgtcccacttccagatgaggcgctggaagcctccaagaaggttgatg
ccgttttgttaggtgctgtgggtggtcctaaatggggtacaggtagtgttagacctgaacaaggtttactaaaaatccgtaaagaacttcaattgtacgccaacttaagaccatgtaactttgcatccgactctcttttagacttatctcca
atcaagccacaatttgctaaaggtactgacttcgttgttgtcagagaattagtgggaggtatttactttggtaagagaaaggaagacgatggtgatggtgtcgcttgggatagtgaacaatacaccgttccagaagtgcaaagaatcacaag
aatggccgctttcatggccctacaacatgagccaccattgcctatttggtccttggataaagctaatgttttggcctcttcaagattatggagaaaaactgtggaggaaaccatcaagaacgaatttcctacattgaaggttcaacatcaat
tgattgattctgccgccatgatcctagttaagaacccaacccacctaaatggtattatcatcaccagcaatatgtttggtgacatcatctccgatgaagcctccgttatcccaggttccttgggtttgttgccatctgcgtccttggcctct
ttgccagacaagaacaccgcatttggtttgtacgaaccatgccacggttctgctccagatttgccaaagaataaggtcaaccctatcgccactatcttgtctgctgcaatgatgttgaaattgtcattgaacttgcctgaagaaggtaaggc
cattgaagatgcagttaacaaactagtttgagccgcttcaattgcggcaagtccccagttttgtttggtactcccctctcgcctcgcgtgtttttgttcctccgaattgtgcatcaccctgtcacatccgtacaccatcggaacactctgct
cctcctcctttctcctcttaattgaacgtatatctaattaccctaataacaagtatcttcaaccatggacaagaagtactccattgggctcgatatcggcacaaacagcgtcggttgggccgtcattacggacgagtacaaggtgccgagca
aaaaattcaaagttctgggcaataccgatcgccacagcataaagaagaacctcattggcgccctcctgttcgactccggggagacggccgaagccacgcggctcaaaagaacagcacggcgcagatatacccgcagaaagaatcggatctgc
tacctgcaggagatctttagtaatgagatggctaaggtggatgactctttcttccataggctggaggagtcctttttggtggaggaggataaaaagcacgagcgccacccaatctttggcaatatcgtggacgaggtggcgtaccatgaaaa
gtacccaaccatatatcatctgaggaagaagcttgtagacagtactgataaggctgacttgcggttgatctatctcgcgctggcgcatatgatcaaatttcggggacacttcctcatcgaggggggacctgaacccagacaacagcgatgtcg
acaaactctttatccaactggttcagacttacaatcagcttttcgaagagaacccgatcaacgcatccggagttgacgccaaagcaatcctgagcgctaggctgtccaaatcccggcggctcgaaaacctcatcgcacagctccctggggag
aagaagaacggcctgtttggtaatcttatcgccctgtcactcgggctgacccccaactttaaatctaacttcgacctggccgaagatgccaagcttcaactgagcaaagacacctacgatgatgatctcgacaatctgctggcccagatcgg
cgaccagtacgcagaccttttttggcggcaaagaacctgtcagacgccattctgctgagtgatattctgcgagtgaacacggagatcaccaaagctccgctgagcgctagtatgatcaagcgctatgatgagcaccaccaagacttgactt
tgctgaaggcccttgtcagacagcaactgcctgagaagtacaaggaaattttcttcgatcagtctaaaaatggctacgccggatacattgacggcggagcaagccaggaggaattttacaaatttattaagcccatcttggaaaaaatggac
ggcaccgaggagctgctggtaaagcttaacagagaagatctgttgcgcaaacagcgcactttcgacaatggaagcatcccccaccagattcacctgggcgaactgcacgctatcctcaggcggcaagaggatttctacccctttttgaaaga
taacagggaaaagattgagaaaatcctcacatttcggataccctactatgtaggcccccctcgcccggggaaattccagattcgcgtggatgactcgcaaatcagaagagactatcactccctggaacttcgaggaagtcgtggataagggg
cctctgcccagtccttcatcgaaaggatgactaactttgataaaaatctgcctaacgaaaaggtgcttcctaaacactctctgctgtacgagtacttcacagtttataacgagctcaccaaggtcaaatacgtcacagaagggatgagaaag
ccagcattcctgtctggagagcagaagaaagctatcgtggacctcctcttcaagacgaaccggaaagttaccgtgaaacagctcaaagaggactatttcaaaaagattgaatgtttcgactctgttgaaatcagcggagtggaggatcgctt
caacgcatccctgggaacgtatcacgatctcctgaaaatcattaaagacaaggacttcctggacaatgaggagaacgaggacattcttgaggacattgtcctcacccttacgttgtttgaagatagggagatgattgaagaacgcttgaaaa
cttacgctcatctcttcgacgacaaagtcatgaaacagctcaagaggcgccgatatacaggatgggggcggctgtcaagaaaactgatcaatgggatccgagacaagcagagtggaaagacaatcctggattttcttaagtccgatggattt
gccaaccggaacttcatgcagttgatccatgatgactctctcacctttaaggaggacatccagaaagcacaagtttctggccagggggacagccttcacgagcacatcgctaatcttgcaggtagcccagctatcaaaaagggaatactgca
gaccgttaaggtcgtggatgaactcgtcaaagtaatgggaaggcataagcccgagaatatcgttatcgagatggcccgagagaaccaaactacccagaagggacagaagaacagtagggaaaggatgaagaggattgaagagggtataaaag
aactggggtcccaaatccttaaggaacacccagttgaaaacacccagcttcagaatgagaagctctacctgtactacctgcagaacggcagggacatgtacgtggatcaggaactggacatcaatcggctctccgactacgacgtggatcat
atcgtgccccagtcttttctcaaagatgattctattgataataaagtgttgacaagatccgataaaaatagagggaagagtgataacgtcccctcagaagaagttgtcaagaaaatgaaaaattattggcggcagctgctgaacgccaaact
gatcacacaacggaagttcgataatctgactaaggctgaacgaggtggcctgtctgagttggataaagccggcttcatcaaaaggcagcttgttgagacacgccagatcaccaagcacgtggcccaaattctcgattcacgcatgaacacca
agtacgatgaaaatgacaaactgattcgagaggtgaaagttattactctgaagtctaagctcgtctcagatttcagaaaggactttcagttttataaggtgagagagatcaacaattaccaccatgcgcatgatgcctacctgaatgcagtg
gtaggcactgcacttatcaaaaaatatcccaagcttgaatctgaatttgtttacggagactataaagtgtacgatgttaggaaaatgatcgcaaagtctgagcaggaaataggcaaggccaccgctaagtacttcttttacagcaatattat
gaatttttttcaagaccgagattacactggccaatggagagattcggaagcgaccacttatcgaaacaaacggagaaacaggagaaatcgtgtgggacaagggtagggatttcgcgacagtccggaaggtcctgtccatgccgcaggtgaaca
tcgttaaaaagaccgaagtacagaccggaggcttctccaaggaaagtatcctcccgaaaaggaacagcgacaagctgatcgcacgcaaaaaagattgggaccccaagaaatacggcggattcgattctcctacagtcgcttacagtgtactg
gttgtggccaaagtggagaaagggaagtctaaaaaactcaaaagcgtcaaggaactgctgggcatcacaatcatggagcgatcaagcttcgaaaaaaaccccatcgactttctcgaggcgaaaggatataaagaggtcaaaaaagacctcat
cattaagcttcccaagtactctctctttgagcttgaaaacggccggaaacgaatgctcgctagtgcgggcgagctgcagaaaggtaacgagctggcactgccctctaaatacgttaatttcttgtatctggccagccactatgaaaagctca
aagggtcccccgaagataatgagcagaagcagctgttcgtggaacaacacaaacactaccttgatgagatcatcgagcaaataagcgaattctccaaaagagtgatcctcgccgacgctaacctcgataaggtgctttctgcttacaataag
cacagggataagcccatcagggagcaggcagaaaacattatccacttgtttactctgaccaacttgggcgcgcctgcagccttcaagtacttcgacaccaccatagacagaaagcggtacacctctacaaaggaggtcctggacgccacact
gattcatcagtcaattacggggctctatgaaacaagaatcgacctctctcagctcggtggagacagcagggctgaccccaagaagaagaggaaggtgtgagcttcgtccgacggcggcccgacgggtccgaggctgaagcttcgtacgctgc
aggtcgacaacccttaatataacttcgtataatgtatgctatacgaagttattaggtctctggcacgacaggtttcccgactggaaagcgggcagtgagcgcaacgcaattaatgtgagttagctcactcattaggcaccccaggctttaca
ctttatgcttccggctcgtatgttgtgtggaattgtgagcggataacaatttcacacaggaaacagcggcccggagtagtcatacgtgtaggtctttggcgtgatgaaaatcaggtggcgcacgacttttcgtaaagttcgggagggagtgc
tgcaaacggtatataaggaccagtttttctcgcacattatcaattgctctttagtacaaagataatatagggtgttatgagccatattcaacgggaaacgtcgaggccgcgattaaattccaacatggatgctgatttatatgggtataaat
gggctcgcgataatgtcgggcaatcaggtgcgacaatctatcgcttgtatgggaagcccgatgcgccagagttgtttctgaaacatggcaaaggtagcgttgccaatgatgttacagatgagatggtcagactaaactggctgacggaattt
atgcctcttccgaccatcaagcattttatccgtactcctgatgatgcatggttactcaccactgcgatccccggaaaaacagcattccaggtattagaagaatatcctgattcaggtgaaaatattgttgatgcgctggcagtgttcctgcg
ccggttgcattcgattcctgtttgtaattgtccttttaacagcgatcgcgtatttcgtctcgctcaggcgcaatcacgaatgaataacggtttggttgatgcgagtgattttgatgacgagcgtaatggctggcctgttgaacaagtctgga
aagaaatgcataaacttttgccattctcaccggattcagtcgtcactcatggtgatttctcacttgataaccttatttttgacgaggggaaattaataggttgtattgatgttggacgagtcggaatcgcagaccgataccaggatcttgcc
atcctatggaactgcctcggtgagttttctccttcattacagaaacggctttttcaaaaatatggtattgataatcctgatatgaataaattgcagtttcatttgatgctcgatgagtttttctaatcagaattggttaattggttgtaaca
ctggcagagcattacgctgacttgacgggacggcgcaagctcatgaccaaaatcccttaacgtgagttttcgttccactgagcgtcagaccccgtagaaaagatcaaaggatcttcttgagatcctttttttctgcgcgtaatctgctgctt
gcaaacaaaaaaaccaccgctaccagcggtggtttgtttgccggatcaagagctaccaactctttttccgaaggtaactggcttcagcagagcgcagataccaaatactgttcttctagtgtagccgtagttaggccaccacttcaagaact
ctgtagcaccgcctacatacctcgctctgctaatcctgttaccagtggctgctgccagtggcgataagtcgtgtcttaccgggttggactcaagacgatagttaccggataaggcgcagcggtcgggctgaacggggggttcgtgcacacag
cccagcttggagcgaacgacctacaccgaactgagatacctacagcgtgagctatgagaaagcgccacgcttcccgaagggagaaaggcggacaggtatccggtaagcggcagggtcggaacaggagagcgcacgagggagcttccaggggg
aaacgcctggtatctttatagtcctgtcgggtttcgccacctctgacttgagcgtcgatttttgtgatgctcgtcaggggggcggagcctatggaaaaacgccagcaacgcggcctttttacggttcctggccttttgctggccttttgctc
acatgacattaacctataaaaataggcgtatcacgaggc

**Figure S2**. Map and 5'-3' sequence of the pKAM1005 plasmid

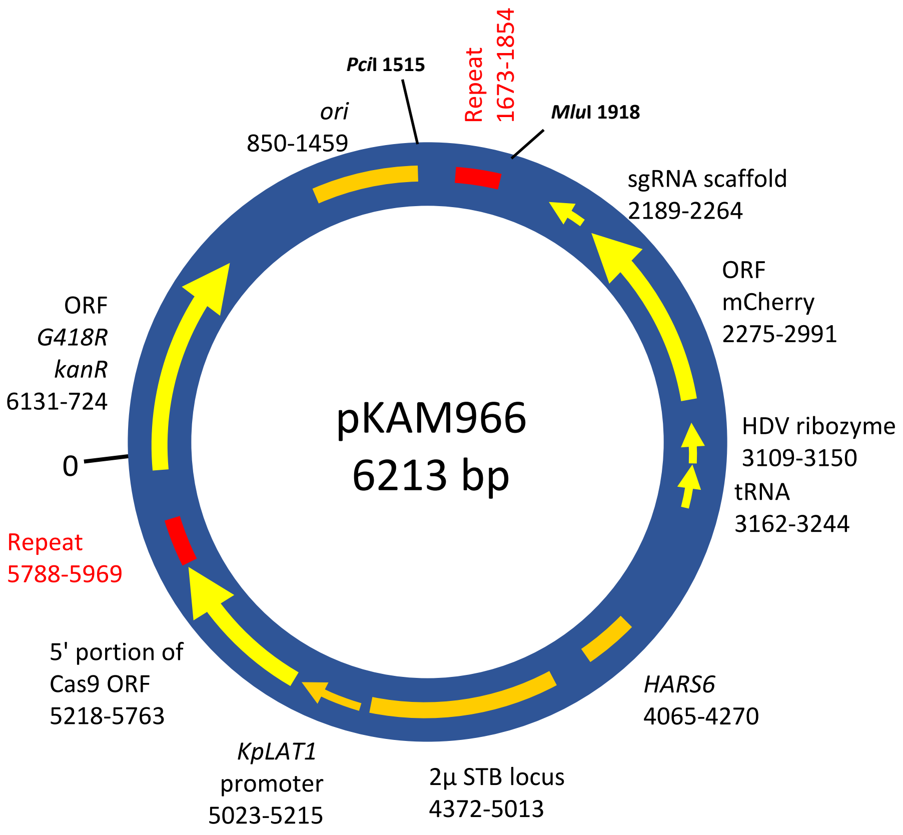


```
>pKAM966
cgataatgtcgggcaatcaggtgcgacaatctatcgcttgtatgggaagcccgatgcgccagagttgtttctgaaacatggcaaaggtagcgttgccaatgatgttacagatgagatggtcagactaaactggctgacggaatttatg
cctcttccgaccatcaagcattttatccgtactcctgatgatgcatggttactcaccactgcgatccccggaaaaacagcattccaggtattagaagaatatcctgattcaggtgaaaatattgttgatgcgctggcagtgttcctgc
gccggttgcattcgattcctgtttgtaattgtccttttaacagcgatcgcgtatttcgtctcgctcaggcgcaatcacgaatgaataacggtttggttgatgcgagtgattttgatgacgagcgtaatggctggcctgttgaacaagt
ctggaaagaaatgcataaacttttgccattctcaccggattcagtcgtcactcatggtgatttctcacttgataaccttatttttgacgaggggaaattaataggttgtattgatgttggacgagtcggaatcgcagaccgataccag
gatcttgccatcctatggaactgcctcggtgagttttctccttcattacagaaacggctttttcaaaaatatggtattgataatcctgatatgaataaattgcagtttcatttgatgctcgatgagtttttctaatcagaattggtta
attggttgtaacactggcagagcattacgctgacttgacgggacggcgcaagctcatgaccaaaatcccttaacgtgagttttcgttccactgagcgtcagaccccgtagaaaagatcaaaggatcttcttgagatcctttttttctg
cgcgtaatctgctgcttgcaaacaaaaaaaccaccgctaccagcggtggtttgtttgccggatcaagagctaccaactctttttccgaaggtaactggcttcagcagagcgcagataccaaatactgttcttctagtgtagccgtagt
taggccaccacttcaagaactctgtagcaccgcctacatacctcgctctgctaatcctgttaccagtggctgctgccagtggcgataagtcgtgtcttaccgggttggactcaagacgatagttaccggataaggcgcagcggtcggg
ctgaacggggggttcgtgcacacagcccagcttggagcgaacgacctacaccgaactgagatacctacagcgtgagctatgagaaagcgccacgcttcccgaagggagaaaggcggacaggtatccggtaagcggcagggtcggaaca
ggagagcgcacgagggagcttccagggggaaacgcctggtatctttatagtcctgtcgggtttcgccacctctgacttgagcgtcgatttttgtgatgctcgtcaggggggcggagcctatggaaaaacgccagcaacgcggcctttt
tacggttcctggccttttgctggccttttgctcacatgttctttcctgcgttatcccctgattctgtggataaccgtattaccgcctttgagtgagctgataccgctcgccgcagccgaacgaccgagcgcagcgagtcagtgagcga
ggaagcggaagagcgcccaatacgcaaaccgcctctccccgcgcgttggccgattcattaatgcagctggcacgacaggtttcccgactggaaagcgggcagtgagcgcaacgcaattaatgtgagttagctcactcattaggcaccc
caggctttacactttatgcttccggctcgtatgttgtgtggaattgtgagcggataacaatttcacacaggaaacagctatgaccatgattacgccaagctatttaggtgacactatagaatactcaagctatgcatccaacgcgtag
tcaatgtacggagttatctcaccttagtgttgttgcggtataatgtctgcagccgctgctgtctccccgactttcgccgtgagatcattagacaggcaggctcttgttatatcttacaagggcatgtccagctacccaatcacgagag
gggtccggcggcagaggcactgcctggaatgtccagctatccactagacagaagtttgcgttccatactgtcagaggtggcattttacataacaatagtgacaaaaaataaaaaaagcaccgactcggtgccactttttcaagttgat
aacggactagccttattttaacttgctatttctagctctaaaactgagaccttacttgtatagttcatccatgccaccagtactatgtctcccctcagccctttcgtattgttcaactatcgtataatcttcattatgtgatgtaata
tcaagttttatatttacattgtaagcacctgggagctggacaggtttcttagctttgtatgtcgttttaacctcagcgtcataatgtccaccatctttcaatttcaatctttgtttaatctcacccttcaaggcaccatcttcagggt
acattcgttctgaggaggcctcccagcccattgtcttttttttgcataaccgggccgtcagatggaaagttagttcctcttaatttgactttgtagataaattcaccatcttgcagggacgaatcctgagtcactgttaccacgccacc
gtcttcgaagttcatcacacgctcccacttaaacccttcgggaaaagataactttagatagtctggaatgtcggcggggtgcttgacataagcctttgacccatacatgaattgagggctaaggatatcccatgcaaatggtaatggg
ccacccttagtaacttttagcttcgcggtttgggttccttcataaggccttccttctccttcaccttctatttcaaattcatgtccatttacagaaccttccatgtgaactttgaatctcatgaactccttaatgattgccatgttgt
cttcttctcctttactgttaattaaccccatgcctaggcatggtttagttcctcaccttgtcgtattatactatgccgatatactatgccgatgattaattgtcaacaccgccctaggtctcaagtcccattcgccacccgaaggtgt
tgcccagccggcgccagcgaggaggctgggaccatgccggccatctgcgaattctcccctcccgggatcgaaccggggaccctgagattccttataccattacagtctcatgctcttccaactgagctaaagagagatttgcttaact
cttcttggaggctgcaatataaaatacagcttaaacaatatcctttactaaaggggagtaaagtaacatcatgacggaggaatagatatgacaagctctttcaacgatcaaactttaaactatacgaagattggaaccgtgatttagt
gaggtcttataagtttagtaatcggcaaccggatttgatcatataagagccattgacaaaacttacgggaatataagtttgcgtggtttattacttaggtacaagaaaatgaacaaagattccccttctcctttagaacatattagaa
actttaggatgctcgcagaagatcgactacgtcgttaaggccgtttctgacagagtaaaattcttgagggaactttcaccattatgggaaatggttcaagaaggtattgacttaaactccatcaaatggtcaggtcattgagtgtttt
ttatttgttgtatttttttttttttagagaaaatcctccaatatataaattaggaatcatagtttcatgattttctgttacacctaactttttgtgtggtgccctcctccttgtcaatattaatgttaaagtgcaattctttttcctt
atcacgttgagccattagtatcaatttgcttacctgtattcctttactatcctcctttttctccttcttgataaatgtatgtagattgcgtatatagtttcgtctaccctatgaacatattccatttgtaatttcgtgtcgtttcta
ttatgaatttcatttataaagtttatgtaccgggccccccctcgaggtcgatcttaggttttgtagttaccggtcgctgtttgatggctatttttatttttgagtttttaataattgtggtaatttttttgaaggtcgcagcaacca
tttctggctctgaaaaggctacaacatatgaccggggccatttagtaatcagcacaattcccttctataatatcaagactcaatgcaactgctagtattaccttcgaatagcaggaattttccgatatccatatggttctcagcctag
cagaaatgtcaccaaataagacttcgtggccaagctggtttaaggcatgcgactgttaatcgcaagatcgacggtatcggtagtatatagagacaaaatagaagaaaccgttcataattttctgaccaatgaagaatcatcaacgcta
tcactttctgttcacaaagtatgcgcaatccacatcggtatagaatataatcggggatgcctttatcttgaaaaaatgcacccgcagcttcgctagtaatcagtaaacgcgggaagtggagtcaggcttttttatggaagagaaaat
agacaccaaagtagccttcttctaaccttaacggacctacagtgcaaaaagttatcaagagactgcattatagagcgcacaaaggagaaaaaaagtaatctaagatgctttgttagaaaaatagcgctctcgggatgcatttttgtag
aacaaaaaagaagtatagattctttgttggtaaaatagcgctctcgcgttgcatttctgttctgtaaaaatgcagctcagattctttgtttgaaaaattagcgctctcgcgttgcatttttgttttacaaaaatgaagcacagattct
tcgttggtaaaatagcgctttcgcgttgcatttctgttctgtaaaaatgcagctcagattctttgtttgaaaaattagcgctctcgcgttgcatttttgttctacaaaatgaagcacagatgcttcgttatcagatccactagtttga
gccgcttcaattgcggcaagtccccagttttgtttggtactcccctctcgcctcgcgtgtttttgttcctccgaattgtgcatcaccctgtcacatccgtacaccatcggaacactctgctcctcctcctttctcctcttaattgaac
gtatatctaattaccctaataacaagtatcttcaaccatggacaagaagtactccattgggctcgatatcggcacaaacagcgtcggttgggccgtcattacggacgagtacaaggtgccgagcaaaaaattcaaagttctgggcaat
accgatcgccacagcataaagaagaacctcattggcgccctcctgttcgactccggggagacggccgaagccacgcggctcaaaagaacagcacggcgcagatatacccgcagaaagaatcggatctgctacctgcaggagatcttta
gtaatgagatggctaaggtggatgactctttcttccataggctggaggagtcctttttggtggaggaggataaaaagcacgagcgcccacccaatctttggcaatatcgtggacgaggtggcgtaccatgaaaagtacccaaccatata
tcatctgaggaagaagcttgtagacagtactgataaggctgacttgcggttgatctatctcgcgctggcgcatatgatcaaatttcggggacacttcctcatcgagggggacctgaacccagacaacagcgatgtcgacgtcatatgg
atccgatatcgccgtggcggccgattcattaatgcagctggcacgacaggtttcccgactggaaagcgggcagtgagcgcaacgcaattaatgtgagttagctcactcattaggcaccccaggctttacactttatgcttccggctcg
tatgttgtgtggaattgtgagcggataacaatttcacacaggaaacagcggcccggagtagtcatacgtgtaggtctttggcgtgatgaaaatcaggtggcgcacgacttttcgtaaagttcgggagggagtgctgcaaacggtatat
aaggaccagtttttctcgcacattatcaattgctctttagtacaaagataatatagggtgttatgagccatattcaacgggaaacgtcgaggccgcgattaaattccaacatggatgctgatttatatgggtataaatgggctcg
```

**Figure S3**. Map and 5'-3' sequence of the pKAM966 plasmid

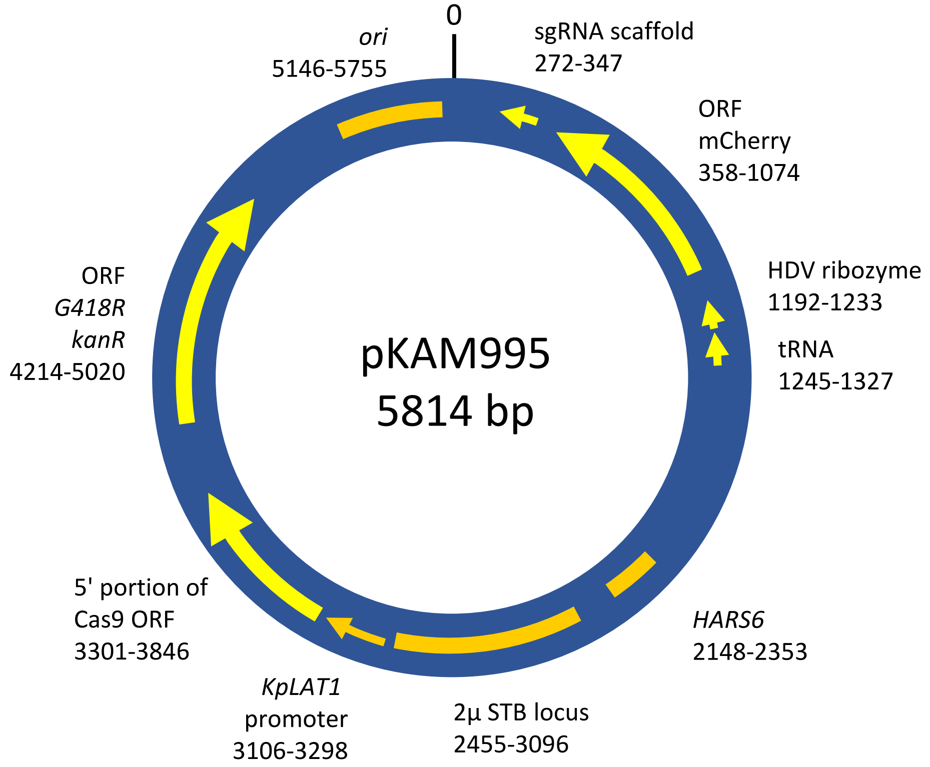


```
>pKAM995
cgcgtagtcaatgtacggagttatctcaccttagtgttgttgcggtataatgtctgcagccgctgctgtctccccgactttcgccgtgagatcattagacaggcaggctcttgttatatcttacaagggcatgtccagctacccaat
cacgagaggggtccggcggcagaggcactgcctggaatgtccagctatccactagacagaagtttgcgttccatactgtcagaggtggcattttacataacaatagtgacaaaaaataaaaaaagcaccgactcggtgccactttttt
caagttgataacggactagccttattttaacttgctatttctagctctaaaactgagaccttacttgtatagttcatccatgccaccagtactatgtctcccctcagccctttcgtattgttcaactatcgtataatcttcattatg
tgatgtaatatcaagttttatatttacattgtaagcacctgggagctggacaggtttcttagctttgtatgtcgttttaacctcagcgtcataatgtccaccatctttcaatttcaatctttgtttaatctcacccttcaaggcacc
atcttcagggtacattcgttctgaggaggcctcccagcccattgtctttttttgcataaccgggccgtcagatggaaagttagttcctcttaatttgactttgtagataaattcaccatcttgcagggacgaatcctgagtcactgt
taccacgccaccgtcttcgaagttcatcacacgctcccacttaaacccttcgggaaaagataactttagatagtctggaatgtcggcggggtgcttgacataagcctttgacccatacatgaattgagggctaaggatatcccatgc
aaatggtaatgggccacccttagtaacttttagcttcgcggtttgggttccttcataaggccttccttctccttcaccttctatttcaaattcatgtccatttacagaaccttccatgtgaactttgaatctcatgaactccttaat
gattgccatgttgtcttcttctcctttactgttaattaaccccatgcctaggcatggtttagttcctcaccttgtcgtattatactatgccgatatactatgccgatgattaattgtcaacaccgccctaggtctcaagtcccattc
gccacccgaaggtgttgcccagccggcgccagcgaggaggctgggaccatgccggccatctgcgaattctcccctcccgggatcgaaccggggaccctgagattccttataccattacagtctcatgctcttccaactgagctaaag
agagatttgcttaactcttcttggaggctgcaatataaaatacagcttaaacaatatcctttactaaaggggagtaaagtaacatcatgacggaggaatagatatgacaagctctttcaacgatcaaactttaaactatacgaagat
tggaaccgtgatttagtgaggtcttataagtttagtaatcggcaaccggatttgatcatataagagccattgacaaaacttacgggaatataagtttgcgtggtttattacttaggtacaagaaaatgaacaaagattccccttctc
ctttagaacatattagaaactttaggatgctcgcagaagatcgactacgtcgttaaggccgtttctgacagagtaaaattcttgagggaactttcaccattatgggaaatggttcaagaaggtattgacttaaactccatcaaatgg
tcaggtcattgagtgtttttttatttgttgtattttttttttttttagagaaaatcctccaatatataaattaggaatcatagtttcatgattttctgttacacctaactttttgtgtggtgccctcctccttgtcaatattaatgtta
aagtgcaattcttttttccttatcacgttgagccattagtatcaatttgcttacctgtattcctttactatcctcctttttctccttcttgataaatgtatgtagattgcgtatatagtttcgtctaccctatgaacatattccattt
tgtaatttcgtgtcgtttctattatgaatttcatttataaagtttatgtaccgggcccccctcgaggtcgatcttaggttttgtagttaccggtcgctgtttgatggctatttttatttttgagtttttaataattgtggtaat
tttttgaaggtcgcagcaaccatttctggctctgaaaaggctacaacatatgaccggggccatttagtaatcagcacaattcccttctataatatcaagactcaatgcaactgctagtattaccttcgaatagcaggaattttccga
tatccatatggttctcagcctagcagaaatgtcaccaaataagacttcgtggccaagctggtttaaggcatgcgactgttaatcgcaagatcgacggtatcggtagtatatagagacaaaatagaagaaaccgttcataattttctg
accaatgaagaatcatcaacgctatcactttctgttcacaaagtatgcgcaatccacatcggtatagaatataatcggggatgcctttatcttgaaaaaatgcacccgcagcttcgctagtaatcagtaaacgcgggaagtggagtc
aggcttttttatggaagagaaaatagacaccaaagtagccttcttctaaccttaacggacctacagtgcaaaaagttatcaagagactgcattatagagcgcacaaaggagaaaaaaagtaatctaagatgctttgttagaaaaat
agcgctctcgggatgcatttttgtagaacaaaaaagaagtatagattctttgttggtaaaatagcgctctcgcgttgcatttctgttctgtaaaaatgcagctcagattctttgtttgaaaaattagcgctctcgcgttgcattttt
gttttacaaaaatgaagcacagattcttcgttggtaaaatagcgctttcgcgttgcatttctgttctgtaaaaatgcagctcagattctttgtttgaaaaattagcgctctcgcgttgcatttttgttctacaaaatgaagcacaga
tgcttcgttatcagatccactagtttgagccgcttcaattgcggcaagtccccagttttgtttggtactcccctctcgcctcgcgtgtttttgttcctccgaattgtgcatcaccctgtcacatccgtacaccatcggaacactctg
ctcctcctcctttctcctcttaattgaacgtatatctaattaccctaataacaagtatcttcaaccatggacaagaagtactccattgggctcgatatcggcacaaacagcgtcggttgggccgtcattacggacgagtacaaggtg
ccgagcaaaaaattcaaagttctgggcaataccgatcgccacagcataaagaagaacctcattggcgccctcctgttcgactccggggagacggccgaagccacgcggctcaaaagaacagcacggcgcagatatacccgcagaaag
aatcggatctgctacctgcaggagatctttagtaatgagatggctaaggtggatgactctttcttccataggctggaggagtccttttttggtggaggaggataaaaagcacgagcgccacccaatctttggcaatatcgtggacgag
gtggcgtaccatgaaaagtacccaaccatatatcatctgaggaagaagcttgtagacagtactgataaggctgacttgcggttgatctatctcgcgctggcgcatatgatcaaatttcggggacacttcctcatcgagggggacctg
aacccagacaacagcgatgtcgacgtcatatggatccgatatcgccgtggcggccgattcattaatgcagctggcacgacaggtttcccgactggaaagcgggcagtgagcgcaacgcaattaatgtgagttagctcactcattagg
caccccaggctttacactttatgcttccggctcgtatgttgtgtggaattgtgagcggataacaatttcacacaggaaacagcggcccggagtagtcatacgtgtaggtctttggcgtgatgaaaatcaggtggcgcacgacttttc
gtaaagttcgggagggagtgctgcaaacggtatataaggaccagtttttctcgcacattatcaattgctctttagtacaaagataatatagggtgttatgagccatattcaacgggaaacgtcgaggccgcgattaaattccaacat
ggatgctgatttatatgggtataaatgggctcgcgataatgtcgggcaatcaggtgcgacaatctatcgcttgtatgggaagcccgatgcgccagagttgtttctgaaacatggcaaaggtagcgttgccaatgatgttacagatga
gatggtcagactaaactggctgacggaatttatgcctcttccgaccatcaagcattttatccgtactcctgatgatgcatggttactcaccactgcgatccccggaaaaacagcattccaggtattagaagaatatcctgattcagg
tgaaaatattgttgatgcgctggcagtgttcctgcgccggttgcattcgattcctgtttgtaattgtccttttaacagcgatcgcgtatttcgtctcgctcaggcgcaatcacgaatgaataacggtttggttgatgcgagtgattt
tgatgacgagcgtaatggctggcctgttgaacaagtctggaaagaaatgcataaacttttgccattctcaccggattcagtcgtcactcatggtgatttctcacttgataaccttatttttgacgaggggaaattaataggttgtat
tgatgttggacgagtcggaatcgcagaccgataccaggatcttgccatcctatggaactgcctcggtgagttttctccttcattacagaaacggctttttcaaaaatatggtattgataatcctgatatgaataaattgcagtttca
tttgatgctcgatgagtttttctaatcagaattggttaattggttgtaacactggcagagcattacgctgacttgacgggacggcgcaagctcatgaccaaaatcccttaacgtgagttttcgttccactgagcgtcagaccccgta
gaaaagatcaaaggatcttcttgagatcctttttttctgcgcgtaatctgctgcttgcaaacaaaaaaaccaccgctaccagcggtggtttgtttgccggatcaagagctaccaactctttttccgaaggtaactggcttcagcaga
gcgcagataccaaatactgttcttctagtgtagccgtagttaggccaccacttcaagaactctgtagcaccgcctacatacctcgctctgctaatcctgttaccagtggctgctgccagtggcgataagtcgtgtcttaccgggttg
gactcaagacgatagttaccggataaggcgcagcggtcgggctgaacggggggttcgtgcacacagcccagcttggagcgaacgacctacaccgaactgagatacctacagcgtgagctatgagaaagcgccacgcttcccgaaggg
agaaaggcggacaggtatccggtaagcggcagggtcggaacaggagagcgcacgagggagcttccagggggaaacgcctggtatctttatagtcctgtcgggtttcgccacctctgacttgagcgtcgatttttgtgatgctcgtca
ggggggcggagcctatggaaaaacgccagcaacgcggcctttttacggttcctggccttttgctggccttttgctcacatg
```

**Figure S4**. Map and 5'-3' sequence of the pKAM995 plasmid

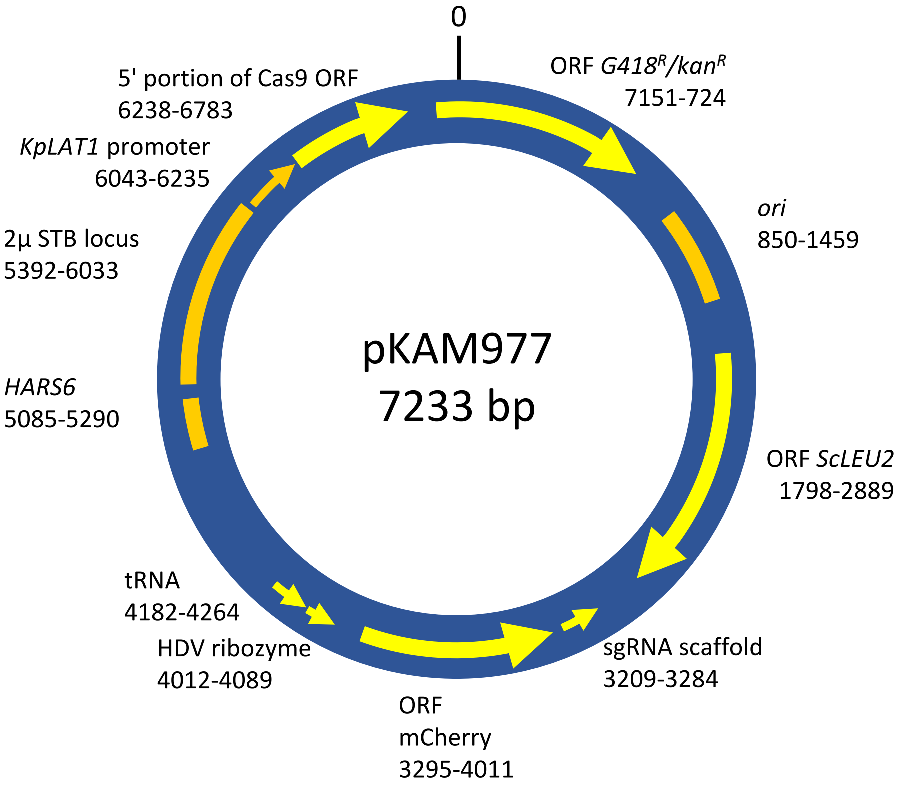


```
>pKAM977
cgataatgtcgggcaatcaggtgcgacaatctatcgcttgtatgggaagcccgatgcgccagagttgtttctgaaacatggcaaaggtagcgttgccaatgatgttacagatgagatggtcagactaaactggctgacggaattta
tgcctcttccgaccatcaagcattttatccgtactcctgatgatgcatggttactcaccactgcgatccccggaaaaacagcattccaggtattagaagaatatcctgattcaggtgaaaatattgttgatgcgctggcagtgttc
ctgcgccggttgcattcgattcctgtttgtaattgtccttttaacagcgatcgcgtatttcgtctcgctcaggcgcaatcacgaatgaataacggtttggttgatgcgagtgattttgatgacgagcgtaatggctggcctgttga
acaagtctggaaagaaatgcataaacttttgccattctcaccggattcagtcgtcactcatggtgatttctcacttgataaccttatttttgacgaggggaaattaataggttgtattgatgttggacgagtcggaatcgcagacc
gataccaggatcttgccatcctatggaactgcctcggtgagttttctccttcattacagaaacggctttttcaaaaatatggtattgataatcctgatatgaataaattgcagtttcatttgatgctcgatgagtttttctaatca
gaattggttaattggttgtaacactggcagagcattacgctgacttgacgggacggcgcaagctcatgaccaaaatcccttaacgtgagttttcgttccactgagcgtcagaccccgtagaaaagatcaaaggatcttcttgagat
cctttttttctgcgcgtaatctgctgcttgcaaacaaaaaaaccaccgctaccagcggtggtttgtttgccggatcaagagctaccaactctttttccgaaggtaactggcttcagcagagcgcagataccaaatactgttcttct
agtgtagccgtagttaggccaccacttcaagaactctgtagcaccgcctacatacctcgctctgctaatcctgttaccagtggctgctgccagtggcgataagtcgtgtcttaccgggttggactcaagacgatagttaccggata
aggcgcagcggtcgggctgaacggggggttcgtgcacacagcccagcttggagcgaacgacctacaccgaactgagatacctacagcgtgagctatgagaaagcgccacgcttcccgaagggagaaaggcggacaggtatccggta
agcggcagggtcggaacaggagagcgcacgagggagcttccagggggaaacgcctggtatctttatagtcctgtcgggtttcgccacctctgacttgagcgtcgatttttgtgatgctcgtcaggggggcggagcctatggaaaaa
cgccagcaacgcggcctttttacggttcctggccttttgctggccttttgctcacatgacattaacctataaaaataggcgtatcacgaggccctttcgtcttcaagaattaactgtgggaatactcaggtatcgtaagatgcaag
agttcgaatctcttagcaaccattatttttttcctcaacataacgagaacacacaggggcgctatcgcacagaatcaaattcgatgactggaaattttttgttaatttcagaggtcgcctgacgcatataccttttttcaactgatt
acatttcagcaatatatatatatatttcaaggatataccattctaatgtctgcccctaagaagatcgtcgttttgccaggtgaccacgttggtcaagaaatcacagccgaagccattaaggttcttaaagctatttctgatgttcg
ttccaatgtcaagttcgatttcgaaaatcatttaattggtggtgctgctatcgatgctacaggtgtcccacttccagatgaggcgctggaagcctccaagaaggttgatgccgttttgttaggtgctgtgggtggtcctaaatggg
gtacaggtagtgttagacctgaacaaggtttactaaaaatccgtaaagaacttcaattgtacgccaacttaagaccatgtaactttgcatccgactctcttttagacttatctccaatcaagccacaatttgctaaaggtactgac
ttcgttgttgtcagagaattagtgggaggtatttactttggtaagagaaaggaagacgatggtgatggtgtcgcttgggatagtgaacaatacaccgttccagaagtgcaaagaatcacaagaatggccgctttcatggccctaca
acatgagccaccattgcctatttggtccttggataaagctaatgttttggcctcttcaagattatggagaaaaactgtggaggaaaccatcaagaacgaatttcctacattgaaggttcaacatcaattgattgattctgccgcca
tgatcctagttaagaacccaacccacctaaatggtattatcatcaccagcaatatgtttggtgacatcatctccgatgaagcctccgttatcccaggttccttgggtttgttgccatctgcgtccttggcctctttgccagacaag
aacaccgcatttggtttgtacgaaccatgccacggttctgctccagatttgccaaagaataaggtcaaccctatcgccactatcttgtctgctgcaatgatgttgaaattgtcattgaacttgcctgaagaaggtaaggccattga
agatgcagttaaaaaggttttggatgcaggtatcagaactggtgatttaggtggttccaacagtaccaccgaagtcggtgatgctgtcgccgaagaagttaagaaaatccttgcttaaaaagattctcttttttttatgatatttgt
accgggccccccctcgacgcgtagtcaatgtacggagttatctcaccttagtgttgttgcggtataatgtctgcagccgctgctgtctccccgactttcgccgtgagatcattagacaggcaggctcttgttatatcttacaaggg
catgtccagctacccaatcacgagaggggtccggcggcagaggcactgcctggaatgtccagctatccactagacagaagtttgcgttccatactgtcagaggtggcattttacataacaatagtgacaaaaaataaaaaaagcac
cgactcggtgccactttttcaagttgataacggactagccttattttaacttgctatttctagctctaaaactgagaccttacttgtatagttcatccatgccaccagtactatgtctcccctcagccctttcgtattgttcaact
atcgtataatcttcattatgtgatgtaatatcaagttttatatttacattgtaagcacctgggagctggacaggtttcttagctttgtatgtcgttttaacctcagcgtcataatgtccaccatctttcaatttcaatctttgttt
aatctcacccttcaaggcaccatcttcagggtacattcgttctgaggaggcctcccagcccattgtcttttttttgcataaccgggccgtcagatggaaagttagttcctcttaatttgactttgtagataaattcaccatcttgca
gggacgaatcctgagtcactgttaccacgccaccgtcttcgaagttcatcacacgctcccacttaaacccttcgggaaaagataactttagatagtctggaatgtcggcggggtgcttgacataagcctttgacccatacatgaat
tgagggctaaggatatcccatgcaaatggtaatgggccacccttagtaacttttagcttcgcggtttgggttccttcataaggccttccttctccttcaccttctatttcaaattcatgtccatttacagaaccttccatgtgaac
tttgaatctcatgaactccttaatgattgccatgttgtcttcttctcctttactgttaattaaccccatgcctaggcatggtttagttcctcaccttgtcgtattatactatgccgatatactatgccgatgattaattgtcaaca
ccgccctaggtctcaagtcccattcgccacccgaaggtgttgcccagccggcgccagcgaggaggctgggaccatgccggccatctgcgaattctcccctcccgggatcgaaccggggaccctgagattccttataccattacagt
ctcatgctcttccaactgagctaaagagagatttgcttaactcttcttggaggctgcaatataaaatacagcttaaacaatatcctttactaaaggggagtaaagtaacatcatgacggaggaatagatatgacaagctctttcaa
cgatcaaactttaaactatacgaagattggaaccgtgatttagtgaggtcttataagtttagtaatcggcaaccggatttgatcatataagagccattgacaaaacttacgggaatataagtttgcgtggtttattacttaggtac
aagaaaatgaacaaagattccccttctcctttagaacatattagaaactttaggatgctcgcagaagatcgactacgtcgttaaggccgtttctgacagagtaaaattcttgagggaactttcaccattatgggaaatggttcaag
aaggtattgacttaaactccatcaaatggtcaggtcattgagtgtttttttatttgttgtattttttttttttttagagaaaatcctccaatatataaattaggaatcatagtttcatgattttctgttacacctaactttttgtgtg
gtgccctcctccttgtcaatattaatgttaaagtgcaattctttttccttatcacgttgagccattagtatcaatttgcttacctgtattcctttactatcctcctttttctccttcttgataaatgtatgtagattgcgtatata
gtttcgtctaccctatgaacatattccattttgtaatttcgtgtcgtttctattatgaatttcatttataaagtttatgtaccgggccccccctcgaggtcgatcttaggttttgtagttaccggtcgctgtttgatggctatttt
ttatttttgagtttttttaataattgtggtaatttttttgaaggtcgcagcaaccatttctggctctgaaaaggctacaacatatgaccggggccatttagtaatcagcacaattcccttctataatatcaagactcaatgcaactgc
tagtattaccttcgaatagcaggaattttccgatatccatatggttctcagcctagcagaaatgtcaccaaataagacttcgtggccaagctggtttaaggcatgcgactgttaatcgcaagatcgacggtatcggtagtatatag
agacaaaatagaagaaaccgttcataattttctgaccaatgaagaatcatcaacgctatcactttctgttcacaaagtatgcgcaatccacatcggtatagaatataatcggggatgcctttatcttgaaaaaatgcacccgcagc
ttcgctagtaatcagtaaacgcgggaagtggagtcaggcttttttatggaagagaaaatagacaccaaagtagccttcttctaaccttaacggacctacagtgcaaaaagttatcaagagactgcattatagagcgcacaaagga
gaaaaaaagtaatctaagatgctttgttagaaaaatagcgctctcgggatgcatttttgtagaacaaaaaagaagtatagattctttgttggtaaaatagcgctctcgcgttgcatttctgttctgtaaaaatgcagctcagattc
tttgtttgaaaaattagcgctctcgcgttgcatttttgttttacaaaaatgaagcacagattcttcgttggtaaaatagcgctttcgcgttgcatttctgttctgtaaaaatgcagctcagattctttgtttgaaaaattagcgct
ctcgcgttgcatttttgttctacaaaatgaagcacagatgcttcgttatcagatccactagtttgagccgcttcaattgcggcaagtccccagttttgtttggtactcccctctcgcctcgcgtgtttttgttcctccgaattgtg
catcaccctgtcacatccgtacaccatcggaacactctgctcctcctccttttctcctcttaattgaacgtatatctaattaccctaataacaagtatcttcaaccatggacaagaagtactccattgggctcgatatcggcacaaa
cagcgtcggttgggccgtcattacggacgagtacaaggtgccgagcaaaaaattcaaagttctgggcaataccgatcgccacagcataaagaagaacctcattggcgccctcctgttcgactccggggagacggccgaagccacgc
ggctcaaaagaacagcacggcgcagatatacccgcagaaagaatcggatctgctacctgcaggagatctttagtaatgagatggctaaggtggatgactctttcttccataggctggaggagtcctttttggtggaggaggataaa
aagcacgagcgccacccaatctttggcaatatcgtggacgaggtggcgtaccatgaaaagtacccaaccatatatcatctgaggaagaagcttgtagacagtactgataaggctgacttgcggttgatctatctcgcgctggcgca
tatgatcaaatttcggggacacttcctcatcgagggggacctgaacccagacaacagcgatgtcgacgtcatatggatccgatatcgccgtggcggccgattcattaatgcagctggcacgacaggtttcccgactggaaagcggg
cagtgagcgcaacgcaattaatgtgagttagctcactcattaggcaccccaggctttacactttatgcttccggctcgtatgttgtgtggaattgtgagcggataacaatttcacacaggaaacagcggcccggagtagtcatacg
tgtaggtctttggcgtgatgaaaatcaggtggcgcacgacttttcgtaaagttcgggagggagtgctgcaaacggtatataaggaccagtttttctcgcacattatcaattgctctttagtacaaagataatatagggtgttatga
gccatattcaacgggaaacgtcgaggccgcgattaaattccaacatggatgctgatttatatgggtataaatgggctcg
```

**Figure S5**. Map and 5'-3' sequence of the pKAM977 plasmid

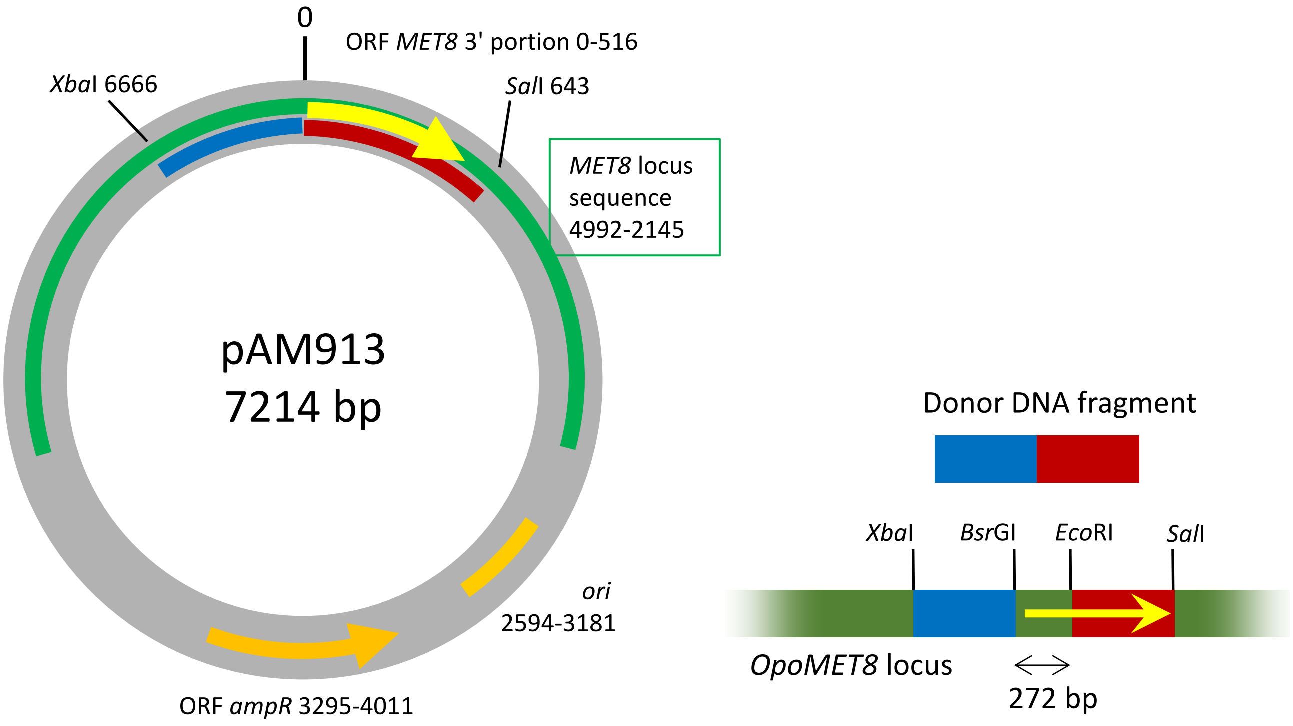


```
>pAM913
aattcagaatggaagatcttgagatgtatcaagaaccaggagacatcagcaagctggatatcaaccacgatcgcgacgaaatagagcgcatttcgagcggtcggtttgctatggtgctcacttgtctcaacaactacgagctgagc
ttacaaatttactatcgctgccggcagctgggcctgaacgttaatcttgctgataaacctaaaaattgtgatttttattttggctctacctatagacagggtccgttacaaataatgatcagctccaatggcaaatcccccggtt
cacaaacagactaaaagaacataagatcaaacccgtgtttgaacaactggacatccggaaagctatagagaaccttgactatctgcgtaccgcgctgcgcaccaagattcaccctggtgagtcaccggaagtgataaagaaccgca
tggagtggaataagaaaatcaccgattcgtattctatcgaggagtggacacaaatggacaaggcccaaattgacagcatcttagcaaactatgctaatgtataatccatcaaactgtctttttcgtttattctacatgtcgtcggt
cttttccgggggcaacatagaagcgtagtgcggcacatacatgtactggccattttggtcgacatattgagactctgctgcgtagggatcgtagaacattggtggacccattccaggcatcatcatacctggtgtttgtgggggcc
cttgagcgggtggctggtgttgaggcatgccaggggcaatgccaggagctgccatcatgtggtttctgttggatcttccccagctgagtctgaccctcgagccgtcgatcacgtatccctggagctgctcgattgcgcgttcggct
tctgtgcgctgcgtgaacttgacaaacccacagcctttcccaggcggcaccttcacatggacaatttggccgtaaggctcgaaaatcgaaagaagagtggcttcggtaatattgtttgacagtccacccacgaagacggtggtatt
tgtaggatcggaataagcaggaggcggttggggcggggcatagtagccaatagggggcattcctggagcgtggtaagggtctagctcaattgggatttggtgtttccggaatagttgattttggtgtttgggagtggccaaagcga
cgcggattagtcttccgtccaaccatttgccgttcatctcgacaagagctttttgccggtcctcgtcgctcgagaatcgcacgaagccaaagcatcgtgacaagccggtggtcgggtcagtcatgactcgcactgttttcaccgtg
ctaaagtgcgtctggaaaagtgcgagcaaatgcgcttcagtagttgcgggtgagaggtctccaacgaaaagcgagtattcaggggtctgggctatctgagagttgagggtcgcggcagatgcccaattgagacggaaaagtttatc
tgttgtgtttggaataatggttccgttgagcttcaacgcctctttcgcgtcttcgggagtctcgaactcaacaaaacaatatccagagtgtgccaattggtgcacaagggcattctggcgaggctttataactttcacaagaacac
gtttatttgttttggcccacacatcggtgataaattcctcgttccaccagggctcgatatcacccatccatagtgtgcgtgagggaactgtatttgcgggattttctggcgggtcgggtagtgtcactggggaattaggttgcggt
gtagggtcggccatatagtggaggcagaggttcgtcaattataaacaactgaaatgtactaaattaagataactctacgtttggttagctaatttttcccgaaattttaacctgtttgctctgcgcggcacaaatcctcgcgctga
ctaaaaatatcgtcgcgcacggaatttgtttatcaactcgtgtttatgctttcacgagaggcaagaaaagaggcaagaaaaagaggctctgccaggaggaaaattgacctcaatggcctcgtgctgaggcccaaagcagagtcgg
ctgggacaagggcgatgcccaagagtcccaaagttttgcctagtcccgtcgcgaacagaactccaagaacaccaaaagctgcgaaaagccccaaaccagagctcgaattaattccctatagtgagtcgtattaaattcgtaatcat
ggtcatagctgtttcctgtgtgaaattgttatccgctcacaattccacacaacatacgagccggaagcataaagtgtaaagcctggggtgcctaatgagtgagctaactcacattaattgcgttgcgctcactgcccgctttccag
tcgggaaacctgtcgtgccagctgcattaatgaatcggccaacgcgcggggagaggcggtttgcgtattgggcgctcttccgcttcctcgctcactgactcgctgcgctcggtcgttcggctgcggcgagcggtatcagctcactc
aaaggcggtaatacggttatccacagaatcaggggataacgcaggaaagaacatgtgagcaaaaggccagcaaaaggccaggaaccgtaaaaaggccgcgttgctggcgtttttccataggctccgcccccctgacgagcatcaca
aaaatcgacgctcaagtcagaggtggcgaaacccgacaggactataaagataccaggcgtttccccctggaagctccctcgtgcgctctcctgttccgaccctgccgcttaccggatacctgtccgcctttctcccttcgggaagc
gtggcgctttctcatagctcacgctgtaggtatctcagttcggtgtaggtcgttcgctccaagctgggctgtgtgcacgaaccccccgttcagcccgaccgctgcgccttatccggtaactatcgtcttgagtccaacccggtaag
acacgacttatcgccactggcagcagccactggtaacaggattagcagagcgaggtatgtaggcggtgctacagagttcttgaagtggtggcctaactacggctacactagaaggacagtatttggtatctgcgctctgctgaagc
cagttaccttcggaaaaagagttggtagctcttgatccggcaaacaaaccaccgctggtagcggtggtttttttgtttgcaagcagcagattacgcgcagaaaaaaaggatctcaagaagatcctttgatcttttctacggggtct
gacgctcagtggaacgaaaactcacgttaagggattttggtcatgagattatcaaaaaggatcttcacctagatccttttaaattaaaaatgaagttttaaatcaatctaaagtatatatgagtaaacttggtctgacagttacca
atgcttaatcagtgaggcacctatctcagcgatctgtctatttcgttcatccatagttgcctgactccccgtcgtgtagataactacgatacgggagggcttaccatctggccccagtgctgcaatgataccgcgagacccacgct
caccggctccagatttatcagcaataaaccagccagccggaagggccgagcgcagaagtggtcctgcaactttatccgcctccatccagtctattaattgttgccgggaagctagagtaagtagttcgccagttaatagtttgcgc
aacgttgttgccattgctacaggcatcgtggtgtcacgctcgtcgtttggtatggcttcattcagctccggttcccaacgatcaaggcgagttacatgatcccccatgttgtgcaaaaaagcggttagctccttcggtcctccgat
cgttgtcagaagtaagttggccgcagtgttatcactcatggttatggcagcactgcataattctcttactgtcatgccatccgtaagatgcttttctgtgactggtgagtactcaaccaagtcattctgagaatagtgtatgcggc
gaccgagttgctcttgcccggcgtcaatacgggataataccgcgccacatagcagaactttaaaagtgctcatcattggaaaacgttcttcggggcgaaaactctcaaggatcttaccgctgttgagatccagttcgatgtaaccc
actcgtgcacccaactgatcttcagcatcttttactttcaccagcgtttctgggtgagcaaaaacaggaaggcaaaatgccgcaaaaaagggaataagggcgacacggaaatgttgaatactcatactcttcctttttcaatatta
ttgaagcatttatcagggttattgtctcatgagcggatacatatttgaatgtatttagaaaaataaacaaataggggttccgcgcacatttccccgaaaagtgccacctgacgcgccctgtagcggcgcattaagcgcggcgggtg
tggtggttacgcgcagcgtgaccgctacacttgccagcgccctagcgcccgctcctttcgctttcttcccttcctttctcgccacgttcgccggctttccccgtcaagctctaaatcggggcatccctttagggttccgatttagt
gctttacggcacctcgaccccaaaaaacttgattagggtgatggttcacgtagtgggccatcgccctgatagacggtttttcgccctttgacgttggagtccacgttctttaatagtggactcttgttccaaactggaacaacact
caaccctatctcggtctattcttttgatttataagggattttgccgatttcggcctattggttaaaaaatgagctgatttaacaaaaatttaacgcgaattttaacaaaatattaacaaaatattaacgtttacaatttcccattc
gccattcaggctgcgcaactgttgggaagggcgatcggtgcgggcctcttcgctattacgccagctggcgaaagggggatgtgctgcaaggcgattaagttgggtaacgccagggttttcccagtcacgacgttgtaaaacgacgg
ccagtgccaagcttctgcagacgcgtcgatcactcgcgcgagttggaacagctgaagaaagaactaagtgatgtcagtgagcaattgagacacaaagagaagaacctgttattatcgagaataaccaaaaagtttcaaacccagtt
taaggagaagttgacagaaatcactggagatccatcaaacgatctctgggatagagtcctgaacgagtttaatcagatcgcacaaagcctagaatcgaaatatgtcttggacgatcaatatgatttcagcttggcatgacagaac
aagaaaacaaggatacacacgctgaattactgcaattgttttggactaagtttaaagacattgctcatgattatgtgacagaagacacagtctcgcgtatcttaagaaacaagtttgaagatctttttagatacgacgataatgac
gtcccaagggtttggctaaatacactggagattgaccagcagtataatagagccagagataacgtgctgagtctgctaccagtattcagcaagatggtgttgactaaaactaatgaggaaattgttccaccaatgaatatttccac
aactgactctgaagagatggatgaagatgataaggagaccacatttgccgagctattatcgacaagacagcagaatggagtactggccagggtcaagaaagagatggatgccatctatatcgaagctaaacgttcggttatggcca
atacgacctcgattcctttctggatgtatgccttggtacttgttttggggtggaatgagtttatggctgttttgcgtaacccattgctgtttatgctgttgattatcctagctacaggggcctattttgctcactatatgggcctg
ctacgtccaatgctgagtgttgcaggggccactttcaaacacaccaagacagtggctaagcaaaaactgcgagagctggtcgaggaggcggaatctgcaaaggcagatgtgaaggcagaaatcgacgaggccaagaccaaataatc
catgtataacttgaaaaaaattagtgtactatatcaaagctgcaaaaataatttaaaaaaattgtttctccctcaccaggacttgaacctggagccttccggttcgtagccgaacgcgataaccattacgctataagggaagaaac
tatcctattattattggatgctattaagtacctcctcgtgctttgattagttgtcaactacttgtttgtattttgattctttaatttcaacggatgcgcttgtcgatcatatgttcgacttattagaagagcagctcgtgcgatca
agcgatacagccttccattacacacactatcgagatgtcccaagtcttttgccgacgtcaaggctgcctcttatagagccatctcttaaaagagaatacttggataaacaccaattatttaccacagatttgttaagtttctttgg
aatttacatgcacaagcctactgggctgctcatatgtgcttgtaaaaacaggaaatcgtttgctctgaggctcgacagatatgagttgcaagatgtcatcaaccgctccgaaagaaatgctctccttttgtccagcaagcagaagg
aaggattacactacataaactataccaatcggccagggttcaagttcagcaagccacatgtagtggtatggtctgatttgcaggaggaaatcactctagaatgctcgaaaagaggactcaaggtagtctacgagatatcgatgcct
gacttcaataatattttcccattgttgcctgtctacttaggcatcagagaaaagggtatagatcgcgccttggatctgtctcaaaatgagtatattcggtatctggcagacggtgagcataaaatcattctatttcagcgactcta
ctcggtacaagaacatttgactttccagctggacggcttgttgcccaacgtgtccaatgctattacggactacttggcgactaaacatcaaggtgatgttgctatctttatcatcgagtggtatcgattgcagctgaaacaaagca
ccaactataaagacgtcgagccaaaggaaactagtgccgacttggctgatttctatggaatcttcccggaagtccttggagacacctcaccggaggccaaagagagcgatttaaatccagacaacgaaatcggtatcacggacgtg
caagtacattccactgatgaccgggtaagcaaatagcaatctggaggtggaaaattgtac
```

**Figure S6**. Map, 5'-3' sequence of the pKAM913 plasmid, and scheme of the donor DNA fragment obtained by cleavage of this plasmid with *Xba*I and *Sal*I. Left and right recombination arms of the donor DNA and homologous sequences in the host genome are show in blue and red, respectively. The sequences of the *OpoMET8* locus unrelated to the recombination arms are shown in green on the scheme. The yellow arrow indicates position of the *OpoMET8* ORF.

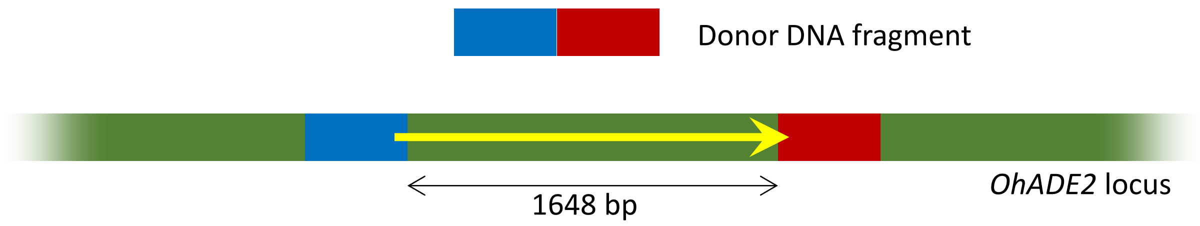


GATGTCGGAAGTCAACAAAATCCAGGAGATCTTCAACGAGAAGAACGACTGCGACTTCTCTACGCTGCCGACGCCTCCTGACGTGCACTCAATCGCTACTCTCTTCAAGAGATATCTGCGAACTCTGAAGGAGACGGTGATCCC
AGAGGAAGAGGCCAAGGAACTCATGTCGCTCACGCTGGCAGCAGATCACGAGGCGTCCGTGCCAAAGGTGAAGGAAACGCTGCAGAAACTGCCAACTTTGAACTTCGACGTTCTCTATGTGCTCTTCAGATACTTCCAGCAGGT
GCTCAAACACAAAGATCTCAACAAGATGAGTATTGGCGCGCTGAGCGTGTTGATGGCTCCCAACCTCACTCCTTTCGATGGTGCCAAGGAGATTTGCTCGGAGCTGCTCACTAATTACAAGTACTACTTTGAGGACGGCGAGAT
AGTGGCGCGCTAGTGCTCCCAGGTCTGGCATTACGTAATCGGAACAATTTTCATAGATAATAATATCCAAGTCAAAAAAATACATTAATTTAATTAGTATCATGGACTCGAAGGTCGTAGGGGCTCTGTTGGCTACGAGGAGTA
TCTTAGAAAATAGATAGGATCGACTTAATTCAATCATCAGCATTTTATTCTGCGAAAAATATAAAATATTTAATTTTTCATGTTCGCCAGTCAGGTCCGATATATTTCCAGATCTCTACGCTTGCACGCCATCAAAGTGCCCGA
TAACGCGCCTACACTCGAGGAAAATGCTGCTTGGCTCAGAAAATTAGGCAGGGCCTCGGGGACACTAAAATCCGCAGGCCCCATAGAATTGCCTAAGTCAACTTACGAGATACGACAAGCTTCGGAGCTCGAGTCCGAAAAAAA
CCCAGAATACATTAACCTAAGGCTTCCTGGGGGCAAACACGTCAAGGTGGTGAAAAAAGCAGACCCACATAGCGAACCAGGAACCTCGTTTTTCACGCTCTCTGTGCCCGCGTCCGACTACTTCGTGGCGTCGTCGCCGTTCTC
GAGACTGCCATCCAACAAACGTCCCAAGGCCTACTCTTCGCAGGCCTTTTCAGACTTGAAAGTCGTTAAGGTGAGGTCGGGAAAGGGTGGAAATGGAG

**Figure S7**. Scheme and 5'-3' sequence of the PCR product used as a donor DNA for obtaining deletion of the *ADE2* gene in *Ogataea haglerorum*. Left and right recombination arms of the donor DNA and homologous sequences in the host genome are show in blue and red, respectively. The sequences of the *OhADE2* locus unrelated to the recombination arms are shown in green on the scheme. The yellow arrow indicates position of the *OhADE2* ORF.
